# Contemporary Clinical Management of the Cerebral Complications of Preeclampsia

**DOI:** 10.1155/2013/985606

**Published:** 2013-12-29

**Authors:** Stefan C. Kane, Alicia Dennis, Fabricio da Silva Costa, Louise Kornman, Shaun Brennecke

**Affiliations:** ^1^Department of Perinatal Medicine, The Royal Women's Hospital, Cnr Grattan Street and Flemington Road, Parkville, VIC 3052, Australia; ^2^Department of Obstetrics and Gynaecology, The University of Melbourne, Parkville, VIC 3010, Australia; ^3^Department of Anaesthetics, The Royal Women's Hospital, Cnr Grattan Street and Flemington Road, Parkville, VIC 3052, Australia; ^4^Department of Obstetrics and Gynaecology and Department of Pharmacology, The University of Melbourne, Parkville, VIC 3010, Australia; ^5^Monash Ultrasound for Women, 15 Murray Street, Clayton, VIC 3170, Australia; ^6^Ultrasound Department, The Royal Women's Hospital, Cnr Grattan Street and Flemington Road, Parkville, VIC 3052, Australia

## Abstract

The neurological complications of preeclampsia and eclampsia are responsible for a major proportion of the morbidity and mortality arising from these conditions, for women and their infants alike. This paper outlines the evidence base for contemporary management principles pertaining to the neurological sequelae of preeclampsia, primarily from the maternal perspective, but with consideration of fetal and neonatal aspects as well. It concludes with a discussion regarding future directions in the management of this potentially lethal condition.

## 1. Scope of the Problem

Preeclampsia (new onset proteinuria and hypertension during pregnancy [1]) is the commonest serious medical disorder of human pregnancy, complicating around 3-4% of pregnancies worldwide [2, 3]. It remains a leading cause of maternal mortality, with 12% of such deaths being attributable to the sequelae of this condition [3], the majority of which occur in the developing world [4]. Even in developed countries, despite an apparent decline in its incidence in some regions [5], preeclampsia is responsible for a significant proportion of maternal deaths: between 2006 and 2008, its mortality rate was 0.83 per 100,000 maternities in the UK, accounting for 18% of direct maternal deaths [6]. Neurological events, such as eclampsia (the pathognomonic convulsive endpoint of preeclampsia) and intracranial haemorrhage, are some of the primary mechanisms by which preeclampsia exerts its fatal maternal influence [7], along with acute pulmonary oedema and hepatic rupture.

In addition to mortality, the maternal morbidity associated with preeclampsia is significant in both the short and long terms. Again, it is the neurological manifestations of this condition that result in a major proportion of this morbidity, including blindness, persistent neurological deficits secondary to stroke, and later cognitive impairment [8]. Effects on the offspring of preeclamptic mothers are no less significant. These infants are commonly born preterm and/or growth restricted, and those who do not succumb to the twofold-increased risk of neonatal mortality [9] are susceptible to long-term neurological disability, as well as cardiovascular and metabolic diseases in later life [10].

The pathogenesis of preeclampsia remains incompletely understood, but is thought to involve a maternal genetic predisposition [11] which leads to defective placentation in early pregnancy, followed by a hyperinflammatory state resulting in widespread endothelial dysfunction [12]. The only curative treatment is delivery of the fetus and placenta [13]. Other current therapies are aimed at the prevention of maternal seizures and severe hypertension, thereby mitigating the effects of this disease without fundamentally altering its course. Trials of therapies that target the pathological processes underlying preeclampsia (e.g., statins [14] and melatonin [15]) are ongoing, with a view to improving neonatal outcome, primarily through prolongation of gestation and improved fetal growth, without unduly compromising maternal health. Similarly, research attention has been paid to identifying agents effective at preventing the development of preeclampsia [16]. Thus far, only aspirin [17] and calcium [18] have demonstrated this benefit, although many other potential agents are under active investigation. Furthermore, the improved prediction of those destined to develop preeclampsia, especially its early onset and severe forms, may lead to better clinical outcomes through the initiation of preventive therapies or enhanced surveillance [19]. Predictive testing strategies using various combinations of maternal factors, serum biomarkers, and ultrasonographic parameters have been studied in the first [20], second [21], and third trimesters [22], with encouraging results.

This paper provides an overview of management principles specific to the neurological complications of preeclampsia. Its focus is primarily on those affecting the woman with this condition, although fetal and neonatal considerations are also briefly addressed.

## 2. Headache/Visual Disturbance

Headache is a relatively common symptom in pregnancy, although its incidence is far greater among those with preeclampsia, with one case-control study having determined an odds ratio of 4.95 (95% CI 2.47–9.92) [23]. No single headache phenotype is typical in preeclampsia: throbbing pain, generalised pressure, or needle-/knife-like sensations have all been reported, although a common attribute is a generally poor response to nonopioid analgesics [24]. Headache is generally considered a premonitory symptom for eclampsia, although it is only present in 56% of patients who develop eclamptic seizures [25], and most preeclamptic patients with headache will not progress to eclampsia. Transcranial Doppler ultrasound studies of the middle cerebral artery in preeclamptic women have demonstrated a strong association between headache and abnormal cerebral perfusion pressure [26]. Adequate control of hypertension may lead to symptomatic improvement in such headaches, although this symptom is also a relatively common side effect of antihypertensive therapy, particularly nifedipine.

The visual disturbance associated with preeclampsia can also take many forms, including scotomata, photopsia, diplopia, blurry vision, and amaurosis fugax [27]. Such symptoms herald seizures in 23% of eclamptic patients [19] and may in part be related to retinal vasospasm [28] and cerebral autoregulatory dysfunction [27]. A wide range of pathologies has been associated with visual disturbance in preeclampsia (relating to different aspects of the visual pathway), including

(i) *cortical blindness*, which affects up to 15% of patients with eclampsia [29] and is thought to be related to the posterior reversible encephalopathy syndrome (see the following). It may rarely be the presenting feature of preeclampsia and generally resolves completely postpartum. Uncommon variants include Balint's syndrome (simultanagnosia, optic ataxia, and ocular apraxia), and Anton syndrome (visual anosognosia) [27];
(ii) * serous retinal detachment* which has been identified in 1–3% of patients with preeclampsia, although it is much more common following eclampsia [30]. It generally resolves spontaneously, with 75% of cases resolving within one week of ophthalmoscopic diagnosis;
(iii) the rare entities of *Purtscher-like retinopathy*, *central retinal vein occlusion*, and* retinal/vitreous haemorrhages*, which have only been associated with preeclampsia in case reports [27].

Assuming that preeclampsia is the cause of a patient's headache and/or visual disturbance, treatment of the former will usually result in resolution of the latter. Atypical presentations, persistent symptomatology, or focal neurological signs should prompt careful consideration of alternative diagnoses, both related to preeclampsia (e.g., intracerebral haemorrhage [31]) and unrelated (e.g., tumour). In these instances, a low threshold for performing neuroimaging and seeking specialist neurological and/or ophthalmological opinion is advisable.

## 3. Eclampsia

Eclampsia is the occurrence of tonic-clonic seizures in pregnancy or the puerperium that cannot be explained by another cause, such as epilepsy—the commonest reason for seizures in pregnant women. Eclamptic convulsions occur in around 2-3% of patients with preeclampsia [5, 32] and may be the presenting feature of this condition. Premonitory symptoms and signs—including headache, visual changes, hypertension, epigastric discomfort, and proteinuria—are present in up to four-fifths of subsequently eclamptic patients [19], although most patients with these features will not fit. Eclampsia remains difficult to predict, as evidenced by the relatively large numbers-needed-to-treat in trials of prophylactic therapy [33]. It remains a potentially lethal complication, with US data indicating a fatality rate of 71.6 per 10,000 cases [7].

### 3.1. Pathogenesis

The pathogenic mechanisms underlying eclamptic seizures remain to be elucidated, although endothelial dysfunction is likely to make a significant contribution [34]. Two theories have been proposed, based on different hypotheses regarding the cerebrovascular response to systemic hypertension:

(i) cerebral “overregulation,” leading to vasospasm, ischaemia, and intracellular (cytotoxic) oedema [35],
(ii) loss of cerebral autoregulation, leading to hyperperfusion, extracellular (vasogenic) oedema [36], and the posterior reversible encephalopathy syndrome (PRES) [34], also known as reversible posterior leukoencephalopathy syndrome (RPLS) [37].

PRES is not unique to either eclampsia or pregnancy and can occur in a wide range of hypertensive states. It is a clinico(neuro)radiological entity typified by the appearance of symmetrical lesions of vasogenic oedema, predominantly in the parietooccipital lobes [38]. Coexistent evidence of ischaemia/infarction has been reported, potentially as a result of vasoconstriction secondary to pressure from oedema [39]. The term PRES is perhaps a misnomer, given that the condition is not always reversible [40] and can affect any part of the brain.

Cerebral autoregulatory dysfunction in preeclampsia has been assessed using MRI [41] and transcranial Doppler ultrasound [26], generally of the middle cerebral artery. A recent cohort study utilised the latter to determine cerebral autoregulation among twenty women with untreated preeclampsia and twenty controls and found that, although the study group had a significantly reduced autoregulation index, there was no correlation between blood pressure or clinical features of disease and impaired cerebral autoregulation [42]. This potentially explains why predicting eclampsia remains challenging.

### 3.2. Prophylaxis

The Magpie (magnesium sulphate for prevention of eclampsia) trial [33] and subsequent meta-analyses [43] have confirmed the superiority of magnesium sulphate (MgSO_4_) over other anticonvulsants in the prevention of eclampsia. Its use halves the rate of eclampsia overall, with a number-needed-to-treat of 63 for women with severe preeclampsia and 109 for those without [33]. Cost-benefit analyses would suggest that maximal utility is achieved through reserving MgSO_4_ for cases of severe preeclampsia [44]. The number-needed-to-treat could only be reduced through better prediction of those destined for eclampsia, which remains an area for further research.

There is no agreement on the optimal dose, timeframe, or route of administration of MgSO_4_, resulting in divergent local policies. The regimen used in the Magpie trial (4 g loading dose followed by 1 g per hour) has the advantage of not requiring assessment of serum magnesium levels, as the risk of toxicity is low and can be predicted by clinical examination [13]. Women with renal impairment do, however, require monitoring of serum levels, and the drug is contraindicated in those with myasthenia gravis. The general safety profile of MgSO_4_ was confirmed in a recent integrative review of use of this agent in (pre)eclampsia, which found low rates of absent patellar reflexes (1.6%), respiratory depression (1.3%), and use of calcium gluconate to reverse the effect of MgSO_4_ (<0.2%), with only one maternal death (in 9556 women) directly attributable to its use [45]. Magnesium sulphate can lower the baseline fetal heart rate and reduce variability on cardiotocography but does not seem to influence the fetal biophysical profile [46] and in fact has neuroprotective effects for the fetus as well.

The mechanism of action of MgSO_4_ in preventing eclampsia is unclear. It may have a direct effect on the cerebrovasculature [47] or may elevate the seizure threshold through membrane stabilisation or other central effects [48]. Given the putative role of impaired cerebral autoregulation in the pathogenesis of eclampsia, it has been postulated that antihypertensive therapy (such as labetalol) could be an effective, more easily administered, and less costly alternative to MgSO_4_ for seizure prophylaxis [49]. Support for this approach is derived from the observation that eclampsia remains a rare event in centres with high utilisation of antihypertensive therapy and minimal use of MgSO_4_, and although pilot trial data were promising [50], adequately powered prospective studies designed to test this hypothesis have proven to be difficult to conduct in the post-Magpie era [51].

### 3.3. Treatment

Eclampsia is an obstetric and medical emergency that necessitates immediate involvement of a consultant-level multidisciplinary team, including obstetricians and obstetric anaesthetists [52], in addition to senior midwifery or obstetric nursing staff. Failure to provide this level of care has consistently been identified in maternal deaths associated with eclampsia [6], and it is inappropriate for more junior staff to make the complex clinical decisions this scenario demands.

The treating team's first priority is supportive care of the fitting woman, with a view to prevent injury and to maintain oxygenation through protection of the airway and application of oxygen by mask. Eclamptic seizures are usually self-limiting, generally lasting only one to two minutes. As with prophylaxis, MgSO_4_ has a clearly established role in the treatment of eclamptic seizures and prevention of their recurrence, having been shown to be superior to both diazepam and phenytoin [53, 54]. Use of magnesium sulphate is associated with a significantly lower rate of recurrent seizures (RR 0.41; 95% CI 0.32–0.51) and lower rate of maternal death (RR 0.62; 95% CI 0.39–0.99) than is achieved with other anticonvulsants [55]. Again, a range of regimens for MgSO_4_ exists: it is generally administered as a loading dose followed by an infusion and is continued for 24 hours postpartum or following the last seizure. Recurrent seizures can be treated with a further bolus of MgSO_4_, necessitating careful attention to the possibility of toxicity. Seizures unresponsive to MgSO_4_ can be treated with benzodiazepines (diazepam or lorazepam) or sodium amobarbital [56] and should raise the prospect of an alternative (or additional) causative pathology.

By definition, eclampsia represents a manifestation of severe preeclampsia, and so assessments of other potential complications of this multisystem disorder must be initiated after the seizure has ceased. Particular attention must be paid to the management of concomitant severe hypertension (see the following), which often (but not always) accompanies eclampsia [57]. Haematological and biochemical tests for preeclampsia should be performed urgently, to establish baseline parameters and assess for disseminated intravascular coagulopathy (DIC), the presence of which requires specialist haematology input. Eclampsia may be complicated by acute pulmonary oedema, and so an in-dwelling catheter should be placed to permit strict fluid balance and pulse oximetry used to identify evolving hypoxaemia.

Fetal bradycardia is common during the eclamptic seizure, followed by a reactive tachycardia on cardiotocography. More concerning fetal heart rate patterns should prompt consideration of abruptio placentae, which occurs in 7–10% of cases [58]. Eclampsia is generally considered an indication for delivery, although this should only occur once the patient is stable, with an adequate airway and oxygenation, controlled seizures, stabilised blood pressure, and treatment of any coagulopathy initiated. These measures also allow for in-utero fetal resuscitation, thereby improving the condition of the infant at delivery. The mode of delivery need not necessarily be caesarean section but should be determined by gestation, cervical favourability, and maternal/fetal status. The risk of intra- and postpartum haemorrhage is increased, especially in the context of DIC or thrombocytopaenia and should be anticipated. Ergometrine and its derivatives should not be used for uterine atony in the patient with preeclampsia, as it can cause severe hypertension and intracranial haemorrhage [6].

Complicating all aspects of the management of eclampsia is morbid obesity, which is strongly associated with preeclampsia [59]. It is incumbent upon hospitals to ensure that clinical infrastructure is adequate for these patients, including bariatric beds and large blood pressure cuffs. Additionally, policies should be implemented that anticipate the potential complications faced by obese pregnant women, who often have difficult airways and intravenous access [60].

After delivery, high dependency care is indicated for all patients after eclampsia, with close monitoring of renal and respiratory function and appropriate referral for psychological support, given the increased risk of postnatal depression and associated psychopathology [61]. Postnatal patients who have been delivered in the context of severe preeclampsia or who have developed this complication after delivery, remain at risk of eclampsia, with 36% of initial eclamptic seizures occurring postpartum [25]. As such, these patients require close clinical observation in the early puerperium and should be treated with prophylactic MgSO_4_ if features premonitory for eclampsia ensue [62]. Neuroimaging is only required for those with an atypical seizure pattern, recurrent seizures, prolonged coma, or focal neurological signs [34].

## 4. Intracranial Haemorrhage/Stroke

The incidence of both ischaemic and haemorrhagic strokes is increased in preeclampsia/eclampsia (OR 4.4, 95% CI 3.6–5.4) [63], with 36% of strokes in pregnancy occurring in women with this concomitant diagnosis [64]. Strokes in women with preeclampsia are more likely to be haemorrhagic [65], with 89% being classified thus in one series [66], and are often (but not always) associated with eclampsia. In addition to permanent neurological deficits, these episodes carry a significant risk of mortality: of the 19 maternal deaths in the UK between 2006 and 2008 attributable to preeclampsia, nine (47%) occurred as a result of intracerebral bleeds [6]. Outcomes of strokes sustained in pregnancy appear to be worse than those in nonpregnant patients, possibly reflecting physiological differences or variations in standards of care [67]. As with eclampsia, the pathogenic processes leading to stroke in preeclampsia are incompletely understood, but are likely to involve endothelial dysfunction and disturbance to cerebral autoregulation [64].

### 4.1. Prevention

The recognition and prompt treatment of severe hypertension in pregnancy remain the mainstay of preventing intracerebral haemorrhage [68]. Failure to provide this care is consistently implicated in otherwise potentially preventable maternal deaths in the context of preeclampsia [6]. Guidelines generally recommend immediate antihypertensive therapy for blood pressures consistently equal to or greater than a systolic of 160 mmHg and/or diastolic of 110 mmHg, equating to a mean arterial pressure (MAP) of around 130 mmHg [69–73]. However, a significant proportion (up to 25% in a US series) [66] of patients may sustain an intracerebral bleed at MAPs lower than 130 mmHg, and there is evolving evidence to suggest that rapidity of change in blood pressure [74], and the absolute level of systolic blood pressure, may be of greater clinical relevance. In light of this, the development of point-of-care tests for the improved identification of those at greatest risk of the neurological effects of hypertension may permit better targeted therapy than that which relies on sphygmomanometry alone.

A range of agents in a variety of preparations is available for the treatment of severe hypertension in pregnancy, including intravenous hydralazine and labetalol and oral labetalol and nifedipine. The Cochrane systematic review of their use in this context found insufficient evidence to recommend one over another, suggesting that choice of agent should be determined by clinician familiarity and side effect profile [75]. The review did, however, recommend against the use of high-dose diazoxide, ketanserin, and nimodipine, and found the antihypertensive effect of magnesium sulphate to be too modest to support its use for this purpose alone. Given regional variations in the availability of these products, local protocols that take these into consideration should be followed, with care taken to avoid “overshoot” hypotension that can lead to abruptio placentae and maternal and fetal compromise.

### 4.2. Diagnosis and Treatment

Strokes may present clinically with headache, altered consciousness, seizures, focal neurology, or visual disturbance [63]. As with eclampsia, a stroke is a clinical emergency. Management is optimised through the early involvement of a senior multidisciplinary team, including neurologists, neurosurgeons, and anaesthetists. The team approach is especially important in the context of the complicating factors of pregnancy and preeclampsia. The primary aims of treatment include preservation of brain tissue, avoidance of further complications (including aspiration and those of preeclampsia), control of blood pressure, and long-term rehabilitation [58]. In the acute phase, the patient requires airway support with maintenance of respiration and positioning to avoid aortocaval compression. Urgent investigations are required to assess for DIC or thrombocytopaenia, which may have contributed to intracranial bleeding.

Where required, intravenous antihypertensive agents should be used to control severe hypertension, with a suggested blood pressure target of ≤160/110 [58]. This is in contrast to ischaemic strokes in the non-pregnant population, in which control of blood pressure is only indicated if severe (≥220/120) or if thrombolysis is to be considered (target ≤180/105) [76, 77]. Control of hypertension in patients with haemorrhagic stroke is necessary to minimise further bleeding, although this benefit must be balanced against the risk of cerebral ischaemia. Evidence for target blood pressure ranges is limited [78], and trials are ongoing in the non-pregnant population to determine optimal blood pressure management in this context [79]. Labetalol has been suggested as the first-line agent for hypertension accompanying stroke in preeclampsia [64], in the light of evidence that it lowers cerebral perfusion pressure without affecting cerebral perfusion [80]. A low threshold should be observed for commencing MgSO_4_ for eclampsia prophylaxis.

Neuroimaging is indicated in all pregnant patients whose clinical condition is suggestive of a cerebrovascular event, with MRI preferred on account of its superior multiplanar resolution and soft-tissue contrast [34]. Such imaging should only be performed once the patient has been stabilised. The timing of delivery will be influenced by fetal condition, gestation and severity of the associated preeclampsia. Choice of mode of delivery requires detailed anaesthetic, neurological, and obstetric input to minimise maternal risk.

Evidence regarding specific treatment strategies for stroke in the context of preeclampsia is limited. Haemorrhagic strokes resulting from ruptured aneurysms or arteriovenous malformations are rare [81] but are amenable to neurosurgical intervention, as are extra-axial haemorrhages secondary to head trauma following eclampsia in the context of coagulopathy. Ischaemic cerebrovascular events are generally treated by anticoagulation, with limited data supporting the safety of thrombolysis in pregnancy [82], especially in the context of coexisting preeclampsia.

In the rehabilitative phase, despite clear evidence of benefit in the non-pregnant population [83], admission to a stroke unit is achieved for only a minority of those with strokes related to pregnancy [84]. Use of such resources may aid in closing the gap in outcomes between these groups.

## 5. Confounders

Pregnancy-related conditions that mimic aspects of preeclampsia may also present with neurological symptoms and signs [85]. For example, TTP-HUS (thrombotic thrombocytopaenic purpura—haemolytic uraemic syndrome) may present with confusion, headache, or seizures [86], and acute fatty liver of pregnancy (AFLP) can be associated with hepatic encephalopathy [87]. Differentiation of these pathologies—although potentially difficult—is important, as specific treatments may be indicated. TTP-HUS does not improve following delivery, whereas preeclampsia does, and hypoglycaemia in the context of deranged liver function tests is suggestive of AFLP.

## 6. Long-Term Maternal Outcomes

The risk of recurrence of preeclampsia in a subsequent pregnancy ranges from 11.5 to 65%, depending on preexisting maternal risk factors and the gestation of disease onset in the prior pregnancy [88]. Women with a history of eclampsia face a 2% overall risk of this complication returning in a subsequent pregnancy, with higher risks for those whose eclampsia was of early onset [89]. The risk of preeclampsia recurring can be reduced by optimising maternal weight and preexisting conditions such as chronic hypertension and diabetes, and commencing calcium supplementation and low-dose aspirin from early gestation in a subsequent pregnancy. Close antenatal surveillance is required for early identification of recurrent preeclampsia, in addition to those complications of which such women remain at risk even in the absence of preeclampsia, such as fetal growth restriction and preterm birth [88].

There is increasing evidence that previously preeclamptic women face increased lifetime risks of ill health, predominantly due to cardiovascular events and metabolic disease. Such women have a relative risk of overall mortality at 14.5 years of 1.49 (95% CI 1.05–2.14), a relative risk of stroke of 1.81 (95% CI 1.45–2.27) after 10.4 years [90], and double the risk of any cerebrovascular event [91]. Later neuroimaging of women with prior preeclampsia [92] and eclampsia [93] demonstrates a greater incidence and severity of cerebral white matter lesions, which have been associated with an increased risk of Alzheimer's disease, vascular dementia, cognitive impairment, and stroke [94]. It is not clear whether preeclampsia simply portends these events, which would have happened anyway, or whether it plays a role in their pathogenesis, although commonality of risk factors for preeclampsia and cardiovascular events (such as obesity, diabetes, and chronic hypertension) and evidence of a shared genetic predisposition [95] suggest a unified causal mechanism. Specific guidelines for the mitigation of these long-term risks in this population are yet to be established, although earlier adoption of proven preventive health strategies would seem reasonable in the meantime.

## 7. Fetal and Neonatal Considerations

As a disease mediated by the placenta, preeclampsia has a significant association with fetal growth restriction and confers a relative risk of 4.2 (95% CI 2.2–8) for delivery of a small-for-gestational-age infant [96]. Overall, up to 12% of fetal growth restriction arises in the context of this maternal diagnosis [97]. Preeclampsia also contributes significantly to rates of preterm birth [32], both spontaneous and iatrogenic on maternal and/or fetal grounds [98]. Growth restriction and prematurity are leading causes of perinatal mortality and morbidity, with neurological disability comprising much of the latter. Additionally, such infants are at increased risk of developing cardiovascular and metabolic disease in later life, as evidenced by the increasing volume of epidemiological data [99] in support of the Barker hypothesis [100].

Given that preeclampsia is cured by delivery, the maternal benefit derived from prolonging such pregnancies is limited to facilitation of transfer to an appropriate care facility and an increased chance of successful induction of labour with advancing gestation. The primary rationale for the expectant management of preeclampsia is to improve neonatal outcomes, by allowing administration of corticosteroids for fetal lung development (if prior to 35 weeks) [101] and achieving greater maturity and growth. Such a policy is generally employed with mild preeclampsia [102], with a randomised trial reporting that delivery ≥37 weeks rather than expectant management beyond this gestation is associated with optimal maternal outcomes without increasing the risk of neonatal complications [103]. A trial to determine the optimal timing of delivery for women with mild preeclampsia between 34 and 37 weeks' gestation is ongoing [104].

Severe preeclampsia is generally regarded as an indication for delivery at any gestation above 34 weeks, although uncertainty remains regarding management at earlier gestations. The Cochrane review of interventionist versus expectant care for severe preeclampsia between 24 and 34 weeks' gestation identified lower rates of neonatal morbidity in pregnancies managed expectantly, although there were insufficient data from which conclusions can be drawn regarding perinatal mortality [105]. In contrast, a subsequent trial involving 264 patients with severe preeclampsia from eight tertiary centres in South America comparing expectant care with delivery following corticosteroid administration at gestations of 28 to 33 weeks found no neonatal or maternal benefit with prolongation of pregnancy, with increased rates of small-for-gestational-age infants and abruptio placentae in this group [106]. These disparate results may reflect variations in care between high and low resource settings [107].

The relationship between fetal exposure to preeclampsia and subsequent development of cerebral palsy is complex. Recent birth registry data from Norway indicate that exposure to preeclampsia is associated with an increased risk of cerebral palsy (OR 2.5, 95% CI 2.0–3.2), mediated through prematurity or being born small-for-gestational-age or both [108]. Among children born at term, preeclampsia is a risk factor for cerebral palsy only among small-for-gestational-age infants. Of note is that normally grown infants delivered before term in the setting of preeclampsia have lower rates of cerebral palsy than infants born prematurely for other reasons, such as intrauterine infection, although these rates are still greater than those for infants born at term [109]. This suggests that, in the absence of growth restriction, preeclampsia is less detrimental to (but not protective of) the fetal brain than other causes of preterm birth [110]. An additional consideration is the fetal neuroprotective effect of maternally administered magnesium sulphate, with early observational data suggesting potential benefit [111] having now been confirmed in a Cochrane review of randomised trials: preterm infants exposed to MgSO_4_ prior to birth have a relative risk of cerebral palsy of 0.68 (95% CI 0.54–0.87), with 63 mothers requiring treatment to avert this outcome in one infant [112]. In light of this evidence, regional guidelines for the use of MgSO_4_ in this context have been developed [113, 114], although further research is required to determine optimal dosage and gestational timeframes [115].

## 8. Future Directions

As this review demonstrates, the implications of preeclampsia can be wide ranging and significant, and much remains to be established about the optimal management of this condition. Research priorities in this area might include:

(i) improved delineation and prediction of the complications of preeclampsia in established disease, especially those of a neurological nature, allowing better targeted maternal therapies;
(ii) an expanded evidence base to support decisions regarding timing of delivery in the fetal interest in preterm preeclampsia; and
(iii) strategies to mitigate the long-term risks of cardiovascular and metabolic diseases in previously preeclamptic women.

Notwithstanding the need for further research, the consistent application of evidence-based management principles outlined in this paper—most of which are simple and relatively inexpensive—would reduce the burden of preeclampsia significantly. Energy expended in discovering new diagnostic and therapeutic strategies for this disease needs to be matched by systematic efforts toward ensuring that existing evidence is applied reliably by all involved in the care of preeclamptic women. Indeed, such an approach is the mainstay of exhortations to improve outcomes in preeclampsia, in both the developed [6] and the developing [116] world alike, and would have a substantial impact on this condition and its potentially devastating consequences—neurological and otherwise.
